# Supplementary material for: Marked T cell activation, senescence, exhaustion and skewing towards TH17 in patients with COVID-19 pneumonia
Source: Nat Commun. 2020 Jul 6;11:3434. doi: 10.1038/s41467-020-17292-4 (PMC7338513; doi:10.1038/s41467-020-17292-4)
Supplement: Supplementary file 1 — Supplementary Information [file 41467_2020_17292_MOESM1_ESM.pdf]

SUPPLEMENTARY INFORMATION

De Biasi S. et al.

Marked T cell activation, senescence, exhaustion and skewing towards TH17 in patients with COVID-19 pneumonia

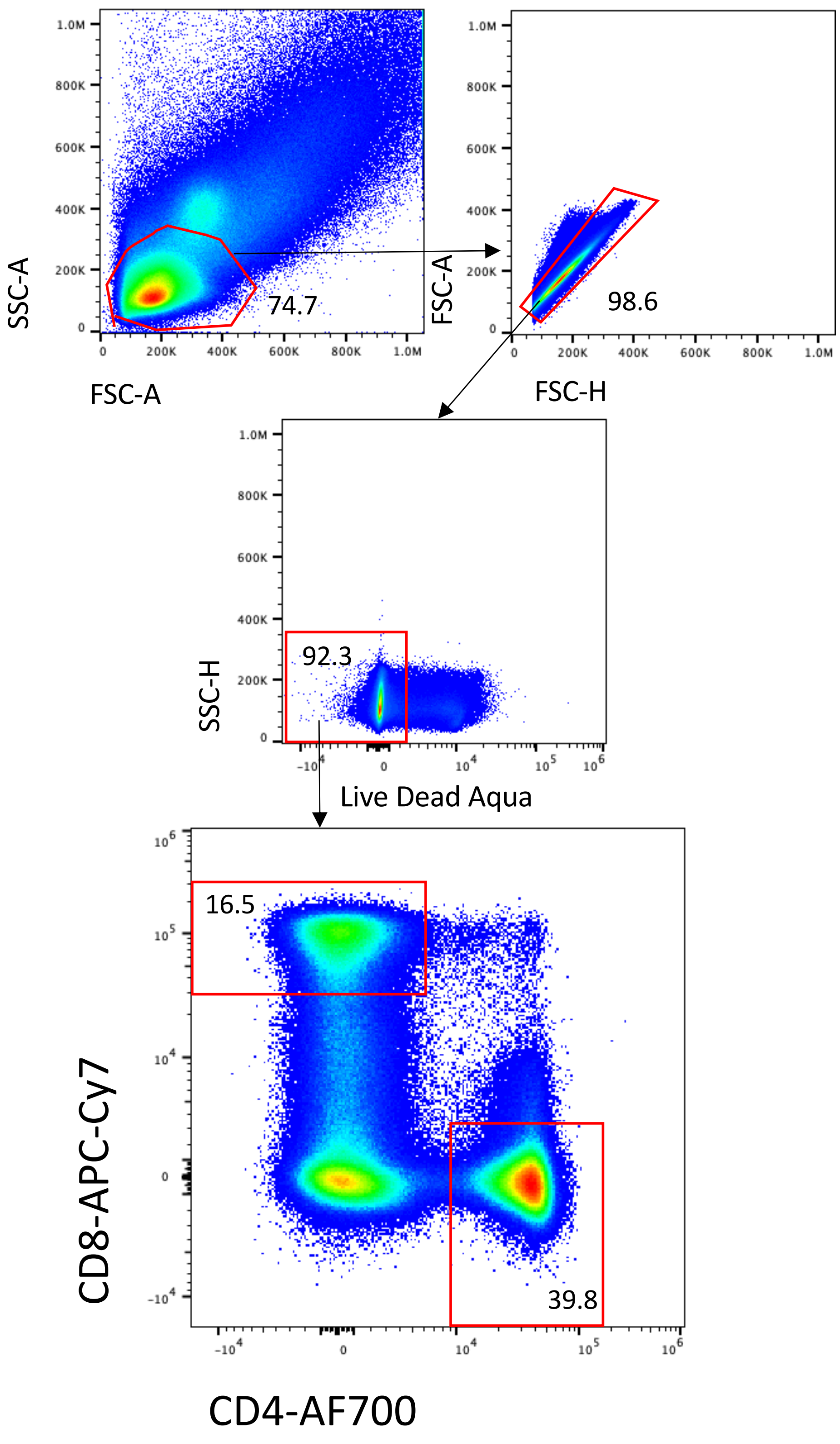

Supplementary Figure 1. Gating strategy used for the characterization of CD4 and CD8 T cells

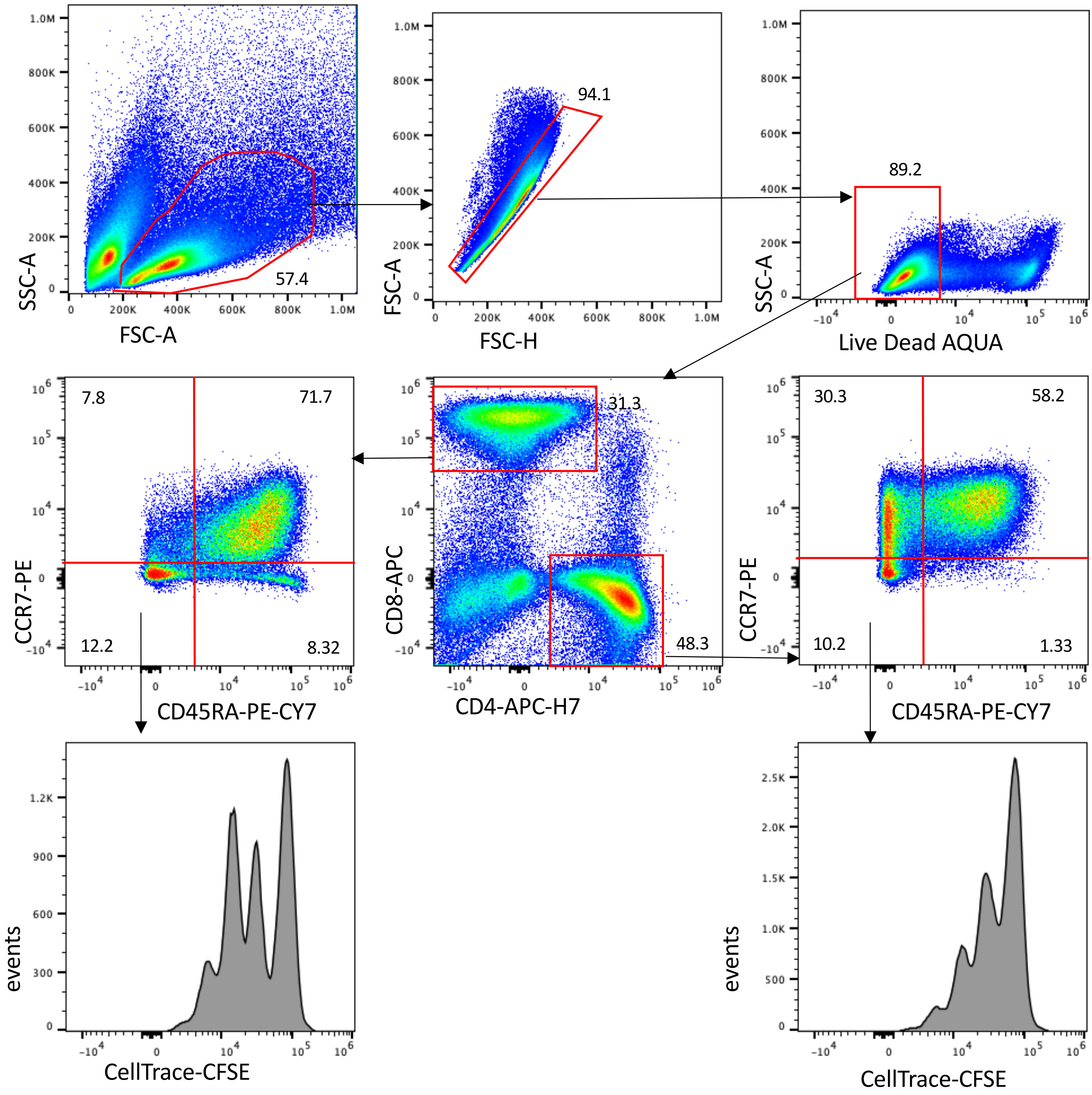

Supplementary Figure 2. Gating strategy used for the analysis of proliferation.

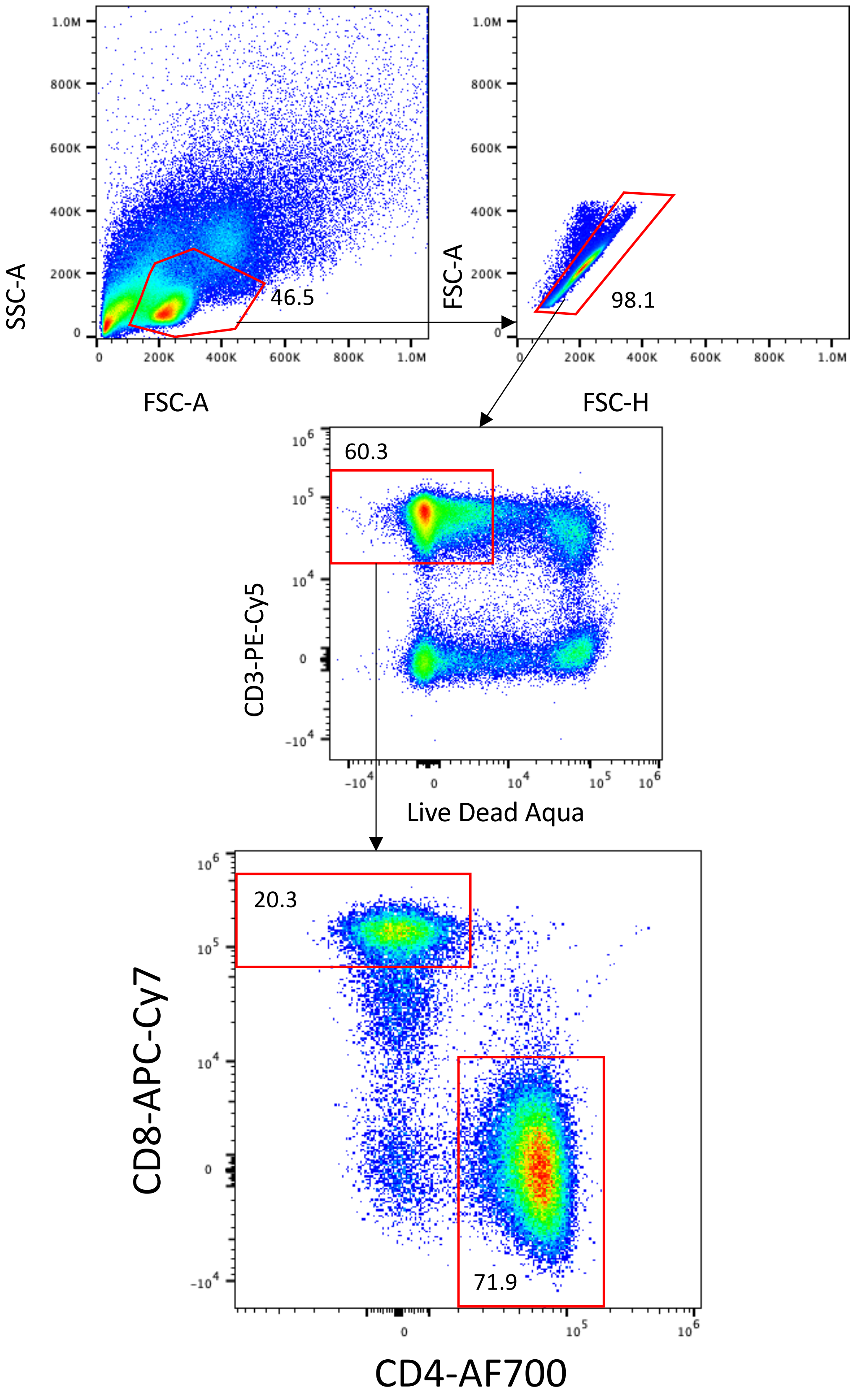

Supplementary Figure 3.
Gating strategy used for the identification of CD4+ and CD8+ T lymphocytes, that was used for the analysis of intracellular cytokines.

Supplementary table 1. CLINICAL CHARACTERISTICS OF THE PATIENTS

PATIENT SYMPTOMS AND TIME CHART

N.	Age (years)	Sex	Type of discharge	Symptoms type - 1	Symptoms type - 2	Days from Admission to Diagnosis	Days from Symptoms to Blood Collection	Days to P/F<250	Days to P/F<150	Days from admission to NIV start
1	57	F	Home	fever	cough	3	11	0	2	2
2	53	M	Home	fever	cough	4	5	4	NA	NA
3	60	M	>30 days in Hospital	fever	cough	-3	10	0	2	2
4	72	M	Home	fever	nausea	1	10	3	3	NA
5	61	M	Home	fever	cough	0	1	1	1	NA
6	65	M	Home	fever	cough	3	19	3	NA	NA
7	75	M	>30 days in Hospital	fever	cough	0	9	0	0	0
8	60	M	Home	fever	cough	0	15	4	5	NA
9	76	M	Death	fever	cough	-2	5	0	0	NA
10	72	M	>30 days in Hospital	dyspnoea		0	9	1	5	8
11	52	M	Home	fever	cough	2	6	1	3	6
12	51	M	Home	fever	dyspnoea	0	9	0	2	NA
13	61	M	Home	fever	dyspnoea	1	2	1	6	2
14	73	M	Death	fever		1	8	1	4	4
15	81	F	>30 days in Hospital	fever	cough	0	5	2	3	3
16	74	M	>30 days in Hospital	fever		-3	4	0	0	1
17	60	M	Home	fever	cough	0	9	NA	NA	NA
18	87	M	Death	fever	dyspnoea	0	2	2	6	NA
19	47	F	Home	fever		0	5	NA	NA	NA
20	36	M	Home	fever		-2	6	NA	NA	NA
21	74	M	Home	fever		0	9	0	0	NA
22	79	M	Home	fever	cough	0	8	2	4	NR
23	40	M	Home	fever	cough	-2	9	2	3	5
24	79	M	Death	dyspnoea	cough	1	0	0	0	0
25	44	M	Home	fever	dyspnoea	-3	4	1	4	NR
26	48	M	Home	fever	cough	-1	11	0	0	NR
27	53	F	Home	fever	cough	-4	7	1	2	NR
28	67	M	Home	fever	dyspnoea	-4	4	0	1	NR
29	67	M	>30 days in Hospital	fever	dyspnoea	1	1	0	0	2
30	59	F	Home	fever	cough	-4	8	2	2	2
31	94	M	>30 days in Hospital	dyspnoea	cough	2	2	1	1	NR
32	85	F	>30 days in Hospital	diarrhoea	nausea	1	3	1	1	NR
33	66	M	Home	fever	cough	-10	8	0	0	1
34	80	F	Home	fever		25	7	0	0	NR
35	83	M	Home	fever	cough	0	7	3	NR	NR
36	72	M	Death	dyspnoea	fever	16	8	NA	NA	20
37	72	M	>30 days in Hospital	dyspnoea		NA	NA	0	0	1
38	40	M	Home	fever		-4	4	NR	NR	NR
39	49	M	>30 days in Hospital	fever	thoracic pain	-2	9	1	3	NR

NA: NOT APPLICABLE
NR: NOT REQUIRED

Supplementary table 2. CLINICAL CHARACTERISTICS OF THE PATIENTS

ARTERIAL BLOOD GAS ANALYSIS AT ADMISSION

N.	pH	pCO2 (mmHg)	pO2 (mmHg)	sO2 (%)	PO2/FiO2 (ratio)
1	7,44	36,4	76	96	238
2	7,42	46,2	64,8	92,6	309
3	7,51	32,3	49,4	90	235
4	7,49	31,5	65,2	94	109
5	7,44	44,4	61,3	93	61
6	7,5	32	115	98,2	411
7	7,44	38,9	62,3	92,8	104
8	7,49	35,9	96,5	97,8	268
9	7,49	32	52,3	91	52
10	7,46	37,3	67,9	94,4	242
11	7,48	37,5	60,2	93,1	286
12	7,47	32,3	115	99	192
13	7,4	30	55,8	92,3	NA
14	7,36	26,7	65,2	91	65
15	7,42	37,3	88,3	96	214
16	7,48	40,1	65,7	94,3	66
17	7,35	36,7	70,9	96	250
18	7,34	44,5	70,9	96	250
19	7,49	42,2	66,7	84	245
20	7,34	34,5	76,7	97,8	330
21	7,54	38,8	71,2	95,2	72
22	7.46	31.6	69.3	94	330
23	7.45	32.7	57.5	91	274
24	7.48	33.0	67.3	94.0	95
25	7.43	39.1	61.8	92.7	294
26	7.40	43.6	66.9	94.2	231
27	7.4	43.5	49.9	86.6	238
28	7.49	33.1	51.8	87.9	247
29	7.45	38.3	58.6	88.9	59
30	7.49	42.5	60.6	93.5	61
31	7 .43	36	64,0	93	106
32	7 .47	27 .5	47 .3	95	82
33	7 .45	37	82,0	NA	119
34	7 .3	81 .8	52 .4	87 .6	90
35	7 .39	29 .8	88 .4	96 .5	421
36	7 .45	29 .5	55 .3	NA	263
37	NA	NA	56 .9	NA	95
38	NA	40 .2	71 .6	94	341
39	7 .47	39 .9	60 .4	93 .3	150

Supplementary table 3. CLINICAL CHARACTERISTICS OF THE PATIENTS

BLOOD PARAMETERS

N.	ALT	Total bilirubin	CK	Creatinine	D-Dimer	Hb	WBC	Neutrophil	Neutrophil	Lymphocytes	Lymphocytes	RBC	INR	LDH	CRP	Platelets
	(U/L)	(mg/L)	(U/L)	(mg/L)	(mg/L)	(g/dL)	(N/uL)	%	(N/uL)	%	(N/uL)	(10^6/L)	(ratio)	(U/L)	(mg/dL)	(10^9/L)
1	75	0.63	35	0.76	650	11	7560	80	6048	18	1361	3.52	1.08	814	19.4	238
2	29	1.02	824	0.63	3410	11.9	5330	74	3944	22	1173	4.03	1.13	1170	6.3	199
3	32	0.76	234	0.75	860	13.7	3470	57	1978	31	1076	4.58	n.a.	814	15.3	158
4	33	0.55	321	0.91	2140	12.9	5470	78	4267	18	985	4.21	1.2	749	7.3	211
5	50	0.65	33	0.93	820	13.9	7730	83	6416	13	1005	4.92	1.09	517	7.2	183
6	38	0.43	570	1.2	1480	14.1	5420	66	3577	22	1192	4.85	1	664	12.8	186
7	40	1.46	305	0.75	1110	13.9	7200	93	6696	5	360	4.25	1	846	22.2	213
8	65	0.6	164	1.07	510	13.7	5600	81	4536	16	896	4.79	1.14	526	5.4	192
9	36	0.34	73	1.77	1220	11.9	4340	87	3776	10	434	4.1	0.89	624	24.7	156
10	24	0.67	96	1.44	780	13.3	NA	NA	NA	NA	NA	NA	1.04	546	7.8	n.a.
11	63	0.76	230	0.98	980	13.6	11010	90	9909	7	771	4.27	1.07	749	20.6	225
12	25	0.41	442	1.17	550	13	5910	83	4905	14	827	4.48	1.18	503	15.2	190
13	23	1.05	183	0.74	720	14.1	5140	78	4009	18	925	4.83	1.01	544	5.3	140
14	29	0.68	555	6.44	40000	11.3	7980	75	5985	21	1676	3.84	1.41	2026	7.9	106
15	24	0.39	206	0.91	2080	12.2	3750	65	2438	30	1125	4.07	0.96	1093	18.7	194
16	56	0.66	38	0.92	8860	12.2	5290	89	4708	18	952	4.1	1.1	1076	2.1	246
17	34	0.78	75	0.78	540	12.5	4350	62	2697	32	1392	4.83	1	650	5.5	250
18	37	0.98	165	0.89	20000	12.5	5520	69	3809	25	1380	3.89	1	760	4.6	230
19	56	0.78	170	0.78	890	12.4	5400	75	4050	21	1134	4.56	1	670	12.3	250
20	245	0.98	200	0.67	560	12.5	4740	50	2370	40	1896	4.56	1.4	780	3.4	245
21	57	0.6	21	0.5	660	12.6	NA	NA	NA	NA	NA	NA	1.12	542	16.8	n.a.
22	28	1.01	NA	1.57	1230	11.4	1330	28	375	61	812	3.73	1.13	683	4.9	81
23	37	0.6	82	0.88	1370	14.4	7380	87	6391	11	841	4.5	1.14	748	24.4	218
24	26	0.81	382	2.15	3430	14.4	8330	79	6547	18	1515	4.72	1.18	762	17.7	106
25	33	0.95	306	1.08	1630	14.9	8410	84	7022	14	1180	4.86	1.1	1093	16.9	258
26	73	0.71	172	0.89	1830	14.1	12780	94	12000	5	663	4.65	1.12	1141	6.5	318
27	23	0.32	52	0.38	650	12	3860	64	2455	31	1194	4.31	0.97	529	12.2	199
28	182	0.46	904	1.36	1210	14.8	4950	82	4079	15	741	4.74	0.95	1134	7.9	167
29	NA	NA	NA	1.06	NA	12.2	6280	84	5300	13	833	4.01	1.1	526	19.3	217
30	28	0.59	234	0.89	870	12.1	7150	79	5613	18	1307	3.95	1.15	733	14.5	191
31	21	0,33	29	1,76	6280	10,5	7400	63	4655	32	2334	3 .35	1 .12	703	4 .9	172
32	20	0,34	NA	0,95	1220	12,9	8020	83	6617	15	1193	4 .71	1 .09	440	16 .5	191
33	170	1,11	NA	0,89	1470	14,5	7980	76	6025	21	1662	4 .57	1 .09	845	19 .2	274
34	23	0,53	59	0,77	1150	13,4	6200	86	5320	12	748	4 .31	1 .03	333	0 .4	257
35	19	<0.3	45	2,58	590	11,3	5440	70	3781	26	1410	3 .37	0 .93	343	6 .3	144
36	5	0,5	<20	1,48	NA	8,6	5050	73	3687	23	1159	3 .29	1 .91	304	10 .3	158
37	21	0,94	349	2,69	NA	16,4	22000	92	20152	7	1571	5 .37	1 .17	1566	48	581
38	47	0,49	50	0,66	300	14,6	5860	65	3832	29	1723	4 .89	0 .97	357	0 .6	203
39	36	1,82	NA	1,38	400	12,8	6470	76	4911	20	1325	4 .42	1 .05	466	8	144

NA: NOT AVAILABLE

Supplementary table 4. CLINICAL CHARACTERISTICS OF THE PATIENTS

CLINICAL PARAMETERS

N.	Fever	Respiratory rate (Breaths/minute)	Systolic blood pressure (mmHg)	Dyastolic blood pressure (mmHg)	Heart rate (beats/minute)	Dyspnoea	Cough	Fatigue	Myalgia	SOFA score2	COPD	diabetes mellitus	hypertension	cardiovascular disease	chronic kidney disease	cancer
1	Yes	30	100	60	82	Yes	Yes	Yes	Yes	2	No	No	Yes	No	No	No
2	no	18	120	90	94	No	Yes	No	No	2	No	Yes	Yes	No	Yes	No
3	Yes	24	130	90	112	No	Yes	Yes	No	3	No	No	Yes	No	No	No
4	Yes	30	120	70	70	Yes	No	Yes	No	2	No	No	Yes	No	No	No
5	Yes	24	118	70	90	No	Yes	No	No	2	No	No	Yes	No	No	No
6	Yes	22	100	60	75	Yes	Yes	Yes	Yes	2	No	No	No	No	No	No
7	no	40	135	90	94	Yes	Yes	No	No	5	No	No	Yes	No	No	No
8	Yes	26	135	70	77	Yes	Yes	No	No	2	No	No	Yes	No	No	No
9	Yes	24	130	70	90	Yes	Yes	Yes	No	4	No	Yes	Yes	Yes	No	No
10	Yes	15	115	60	95	No	Yes	No	No	4	No	No	Yes	No	No	Yes (CLL)
11	Yes	20	120	65	71	No	Yes	Yes	No	1	No	No	No	No	No	No
12	Yes	18	130	75	80	Yes	Yes	No	No	2	No	No	No	No	No	No
13	Yes	33	120	70	89	Yes	No	Yes	No	3	No	No	No	No	No	No
14	Yes	33	110	70	83	Yes	Yes	Yes	No	4	No	No	Yes	Yes	No	No
15	Yes	34	115	60	80	Yes	No	Yes	No	3	No	No	Yes	No	No	No
16	Yes	32	190	95	140	Yes	Yes	Yes	No	2	No	No	Yes	No	No	No
17	Yes	25	130	80	78	Yes	No	Yes	No	2	No	No	No	No	No	No
18	Yes	23	160	80	89	Yes	Yes	Yes	No	3	Yes	No	Yes	No	No	No
19	Yes	23	120	80	89	Yes	Yes	No	No	2	No	No	No	No	No	No
20	Yes	18	120	70	85	No	No	No	No	1	No	No	No	No	No	No
21	Yes	30	200	80	66	Yes	Yes	Yes	Yes	2	Yes	Yes	Yes	No	No	Yes (CLL)
22	No	20	120	80	84	No	Yes	No	No	4	No	No	Yes	No	No	No
23	No	20	105	60	80	Yes	Yes	No	No	2	No	No	Yes	No	No	No
24	No	29	120	80	75	Yes	Yes	No	No	7	No	No	Yes	No	Yes	No
25	No	22	128	75	68	Yes	Yes	No	No	2	No	No	No	No	No	No
26	Yes	35	130	80	112	Yes	Yes	No	No	2	No	No	No	No	No	No
27	Yes	24	122	80	70	Yes	Yes	No	Yes	2	No	Yes	Yes	No	No	No
28	Yes	20	100	70	60	Yes	Yes	No	No	3	No	No	No	No	No	No
29	Yes	20	140	60	88	Yes	Yes	No	No	4	Yes	No	Yes	Yes	No	No
30	No	24	128	60	91	No	Yes	No	No	4	No	No	Yes	No	No	No
31	No	22	NA	NA	NA	Yes	Yes	No	No	4	No	No	Yes	Yes	Yes	No
32	Yes	21	120	80	85	No	No	Yes	Yes	4	No	No	No	No	No	No
33	No	30	146	90	90	Yes	Yes	Yes	No	3	No	No	No	No	No	No
34	No	22	130	80	86	No	No	No	No	4	Yes	No	No	Yes	No	No
35	Yes	24	180	80	90	No	Yes	Yes	No	3	No	Yes	Yes	Yes	Yes	No
36	No	NA	150	70	93	Yes	No	No	No	2	No	No	Yes	Yes	Yes	No
37	Yes	>20	NA	NA	NA	Yes	No	No	No	6	No	Yes	Yes	Yes	Yes	No
38	No	18	125	80	64	Yes	No	No	Yes	1	No	No	No	No	No	No
39	Yes	30	135	70	61	No	No	No	No	6	No	No	No	No	No	No

CLL: CHRONIC LYMPHOCYTIC LEUKEMIA

Supplementary Table 5. List of monoclonal antibodies and reagents used in the study.

Specificity	Fluorochrome	Clone	Manufacturer	Cat. #	Panel	Titer used (µl)
CD3	PE-Cy5	UCHT1	BioLegend	300410	Polyfunctionality	0.6
CD4	AF700	RPA-T4	BioLegend	300526	Polyfunctionality	0.6
CD8	APC-Cy7	RPA-T8	BioLegend	301016	Polyfunctionality	0.6
Granzyme B	BV421	QA18A28	BioLegend	396414	Polyfunctionality	2.5
CD107a	PE	H4A3	BioLegend	328608	Polyfunctionality	0.3
TNF	BV605	MAb11	BioLegend	502936	Polyfunctionality	3.75
IL-17A	PE-Cy7	BL168	BioLegend	512315	Polyfunctionality	3.75
INFg	FITC	B27	BioLegend	506504	Polyfunctionality	2.5
IL-2	APC	MQ1-17H12	BioLegend	500310	Polyfunctionality	2.5
LIVE DEAD	Aqua	N.A.	ThermoFisher	L34966	Polyfunctionality	1.25
CD45RA	FITC	2H4	Beckman Coulter (DuraClone IM T)	B53328	T cell Phenotype	-
CCR7	PE	G043H7	Beckman Coulter (DuraClone IM T)	B53328	T cell Phenotype	-
CD28	ECD	CD28.2	Beckman Coulter (DuraClone IM T)	B53328	T cell Phenotype	-
PD1	PC5.5	PD1.3.5	Beckman Coulter (DuraClone IM T)	B53328	T cell Phenotype	-
CD27	PC7	1A4.CD27	Beckman Coulter (DuraClone IM T)	B53328	T cell Phenotype	-
CD4	APC	13B8.2	Beckman Coulter (DuraClone IM T)	B53328	T cell Phenotype	-
CD8	A700	B9.11	Beckman Coulter (DuraClone IM T)	B53328	T cell Phenotype	-
CD3	APC-A750	UCHT-1	Beckman Coulter (DuraClone IM T)	B53328	T cell Phenotype	-
CD57	Pacific Blue	NC1	Beckman Coulter (DuraClone IM T)	B53328	T cell Phenotype	-
CD45	Krome Orange	J33	Beckman Coulter (DuraClone IM T)	B53328	T cell Phenotype	-
PromoFluor840	Maleimide		Promocell	PK-PF840-3-01	T cell Phenotype	0.3
CD25	BV785	BC96	BioLegend	B302638	T cell Phenotype	1.25
CD127	BV650	A019D5	BioLegend	B351326	T cell Phenotype	0.6
CD95	BUV395	DX2	Becton Dickinson	740306	T cell Phenotype	0.6
CD38	BUV496	HIT2	Becton Dickinson	564658	T cell Phenotype	1.25
HLA-DR	BUV661	G46-6	Becton Dickinson	565073	T cell Phenotype	0.3
CD161	PC7	191B8	Beckman Coulter	B30631	T cell Phenotype	2.5
CCR6	BV605	G034E3	Biolegend	353420	T cell Phenotype	2.5
CCR4	PE-CF594	1G1	Becton Dickinson	565391	T cell Phenotype	2.5
CXCR4	PE	12G5	Biolegend	306506	T cell Phenotype	2.5
TBET	APC	4B10	Biolegend	644814	T cell Phenotype	2.5
GATA3	BV421	16E10A23	Biolegend	653814	T cells Phenotype	5
