# Supplementary material for: Marked T cell activation, senescence, exhaustion and skewing towards TH17 in patients with COVID-19 pneumonia
Source: Nat Commun. 2020 Jul 6;11:3434. doi: 10.1038/s41467-020-17292-4 (PMC7338513; doi:10.1038/s41467-020-17292-4)
Supplement: Supplementary file 3 — Reporting Summary [file 41467_2020_17292_MOESM3_ESM.pdf]

natureresearch

Corresponding author(s): Andrea Cossarizza

Last updated by author(s): June 22, 2020

Reporting Summary

Nature Research wishes to improve the reproducibility of the work that we publish. This form provides structure for consistency and transparency in reporting. For further information on Nature Research policies, see Authors & Referees and the Editorial Policy Checklist.

Statistics

For all statistical analyses, confirm that the following items are present in the figure legend, table legend, main text, or Methods section.

n/a	Confirmed	
☐	☒	The exact sample size (*n*) for each experimental group/condition, given as a discrete number and unit of measurement
☐	☒	A statement on whether measurements were taken from distinct samples or whether the same sample was measured repeatedly
☐	☒	The statistical test(s) used AND whether they are one- or two-sided<br>*Only common tests should be described solely by name; describe more complex techniques in the Methods section.*
☒	☐	A description of all covariates tested
☒	☐	A description of any assumptions or corrections, such as tests of normality and adjustment for multiple comparisons
☐	☒	A full description of the statistical parameters including central tendency (e.g. means) or other basic estimates (e.g. regression coefficient) AND variation (e.g. standard deviation) or associated estimates of uncertainty (e.g. confidence intervals)
☒	☐	For null hypothesis testing, the test statistic (e.g. *F*, *t*, *r*) with confidence intervals, effect sizes, degrees of freedom and *P* value noted<br>*Give P values as exact values whenever suitable.*
☒	☐	For Bayesian analysis, information on the choice of priors and Markov chain Monte Carlo settings
☒	☐	For hierarchical and complex designs, identification of the appropriate level for tests and full reporting of outcomes
☒	☐	Estimates of effect sizes (e.g. Cohen's *d*, Pearson's *r*), indicating how they were calculated

Our web collection on statistics for biologists contains articles on many of the points above.

Software and code

Policy information about availability of computer code

Data collection	Data were coded and recorded in Excel 14.1.0 for Mac in a database present in the Infectious Disease Clinics and routinely used.
Data analysis	For the classical statistical analysis, we used Graph Pad-Prism Vers. 6.0. Concerning the quantification of cytokines, when have not considered in the analysis the values that were above the maximum of the range of detection. For those few values that were below the minimum, we have used the value in the middle between zero and the minimum of the range. When too many values were below the limit of detection, we did not consider the whole measure of the cytokine. For the representation of high parameter flow cytometry Flow Cytometry Standard (FCS) 3.0 files were imported into FlowJo software version 9, and analyzed by standard gating to remove aggregates and dead cells, and identify CD3+ CD4+ T cells and CD8+ T cells. Then, 5,000 CD4+ T cells and 2,500 CD8+ T cells per sample were exported for further analysis in R, by following a script that makes use of Bioconductor libraries and R statistical packages (CATALYST 1.10.1). The script is available at: https://github.com/HelenaLC/CATALYST). The selection of cofactor for data transformation was checked on Cytobank premium version (see: cytobank.org). FlowSOM (available at: https://bioconductor.org/packages/release/bioc/html/FlowSOM.html) was used to perform the metaclustering (K=20); then, data were represented by Uniform Manifold Approximation and Projection (UMAP) method. Concerning intracellular cytokine staining, quantitative variables were compared by Mann-Whitney was applied. We then used Simplified Presentation of Incredibly Complex Evaluation (SPICE) software (version 6, kindly provided by Dr. Mario Roederer, Vaccine Research Center, NIAID, NIH, Bethesda, MD, USA) to investigate polyfunctionality. Cytobank is a platform online (recently bought by Beckman Coulter); FlowSOM is included in Catalyst package.

For manuscripts utilizing custom algorithms or software that are central to the research but not yet described in published literature, software must be made available to editors/reviewers. We strongly encourage code deposition in a community repository (e.g. GitHub). See the Nature Research guidelines for submitting code & software for further information.

Data

Policy information about availability of data

All manuscripts must include a data availability statement. This statement should provide the following information, where applicable:

- Accession codes, unique identifiers, or web links for publicly available datasets
- A list of figures that have associated raw data
- A description of any restrictions on data availability

The data sets generated during the current study are available from the corresponding author on reasonable request. The source data underlying Figures 1, 2, 3, 4 and 7 are provided as a Source Data file. Original .fcs files concerning cytofluorimetric analysis (Figures 1-4) are deposited at the flowrepository.org (55) in the following folders: T cell characterisation: https://flowrepository.org/id/FR-FCM-Z2N5; T cell phenotype: https://flowrepository.org/id/FR-FCM-Z2N4.

Field-specific reporting

Please select the one below that is the best fit for your research. If you are not sure, read the appropriate sections before making your selection.

☒ Life sciences ☐ Behavioural & social sciences ☐ Ecological, evolutionary & environmental sciences

For a reference copy of the document with all sections, see nature.com/documents/nr-reporting-summary-flat.pdf

Life sciences study design

All studies must disclose on these points even when the disclosure is negative.

Sample size

First, in March 2020 we analyzed all the possible patients who came to our observation (n=21). Then we collected anoter group of 18 patients. A post-hoc analysis on sample size was perfomed considering the changes in T cell phenotype and cytokine plasma levels. Considering a cytokine like IL-6 (normal values: mean +/- SD: 1.5 +/- 1.0) with an increase of 50% in patients (which is largely underestimated), 14 patients are sufficient to have an alpha value of 0.05 and a power of 80%. Should the increases be of the order of 300% (as in the case of most cytokines), even 4 COVID patients are sufficient for reaching a power of 90%. Similar considerations are valid for activation markers present in T cells. In this case, we took advantage from our previous experience concerning T cell analysis in patients with HIV, where, for example, a group of 6 subjects with high CD4+ T cells were sufficient to find significant differences in T cell activation or in the plasma level of a cytokine like IL-7 vs. controls (Mussini et al., AIDS 16:1609-1616, 2002).

Data exclusions

For a few cytokines, the Luminex assay gave values that were out of range (OOR). When the OOR were more than 50% of the number of samples, even in one group only, we decided to exclude the cytokine from the analysis. For all details, see “source data” file.

Replication

The values of cytokines are the mean of a technical replicate, and we used the mean. In almost all cases replicates were very similar. Concerning cytofluorimetric analysis, they were performed on several thousand cells according to the Guidelines for the use of Cytometry and Cell Sorting in Immunology (Cossarizza et al., Eur J Immunol. 49: 1457-1973, 2019). As an internal control, In some cases we analyzed the same samples twice or we splitted one sample into two parts, obtaining identical data.

Randomization

We enrolled 39 patients with pneumonia caused by Covid, who had been admitted to the Infectious Diseases Clinics of the University Hospital in Modena, North Italy. They were randomly selected among those who had to start a therapy with anti-IL6R drugs (tocilizumab) because of the severity of their symptoms.

Blinding

For obvious reasons, all blood from human beings is considered infected and treated as such, so rearchers utilized the same procedures for all human samples that we routinely study. So, samples were coded in the Infectious Diseases Clinics and taken to the lab, where blood was treated, stored and subsequently analyzed in a blind manner. Moreover, we applied unsupervised statistical analysis to avoid any possible influence of the operator. The senior authors of the paper (Mussini and Cossarizza) did not open the key until the end of the study.

Reporting for specific materials, systems and methods

We require information from authors about some types of materials, experimental systems and methods used in many studies. Here, indicate whether each material, system or method listed is relevant to your study. If you are not sure if a list item applies to your research, read the appropriate section before selecting a response.

Materials & experimental systems

n/a	Involved in the study	
☐	☒	Antibodies
☒	☐	Eukaryotic cell lines
☒	☐	Palaeontology
☒	☐	Animals and other organisms
☐	☒	Human research participants
☐	☒	Clinical data

Methods

n/a	Involved in the study	
☒	☐	ChIP-seq
☐	☒	Flow cytometry
☒	☐	MRI-based neuroimaging

Antibodies

Antibodies used

Specificity Fluorochrome Clone Manufacturer Cat. # Panel

CD3 PE-Cy5 UCHT1 BioLegend 300410 Polyfunctionality
CD4 AF700 RPA-T4 BioLegend 300526 Polyfunctionality
CD8 APC-Cy7 RPA-T8 BioLegend 301016 Polyfunctionality
Granzyme B BV421 QA18A28 BioLegend 396414 Polyfunctionality
CD107a PE H4A3 BioLegend 328608 Polyfunctionality
TNFα BV605 MAb11 BioLegend 502936 Polyfunctionality
IL-17A PE-Cy7 BL168 BioLegend 512315 Polyfunctionality
INFγ FITC B27 BioLegend 506504 Polyfunctionality
IL-2 APC MQ1-17H12 BioLegend 500310 Polyfunctionality
LIVE DEAD Aqua ThermoFisher L34966 Polyfunctionality
CD45RA FITC 2H4 Beckman Coulter (DuraClone IM T) B53328 T cell Phenotype
CCR7 PE G043H7 Beckman Coulter (DuraClone IM T) B53328 T cell Phenotype
CD28 ECD CD28.2 Beckman Coulter (DuraClone IM T) B53328 T cell Phenotype
PD1 PC5.5 PD1.3.5 Beckman Coulter (DuraClone IM T) B53328 T cell Phenotype
CD27 PC7 1A4.CD27 Beckman Coulter (DuraClone IM T) B53328 T cell Phenotype
CD4 APC 13B8.2 Beckman Coulter (DuraClone IM T) B53328 T cell Phenotype
CD8 A700 B9.11 Beckman Coulter (DuraClone IM T) B53328 T cell Phenotype
CD3 APC-A750 UCHT-1 Beckman Coulter (DuraClone IM T) B53328 T cell Phenotype
CD57 Pacific Blue NC1 Beckman Coulter (DuraClone IM T) B53328 T cell Phenotype
CD45 Krome Orange J33 Beckman Coulter (DuraClone IM T) B53328 T cell
Phenotype PromoFluor840 Maleimide Promocell PK-PF840-3-01 T cell Phenotype
CD25 BV785 BC96 BioLegend B302638 T cell Phenotype
CD127 BV650 A019D5 BioLegend B351326 T cell Phenotype
CD95 BUV395 DX2 Becton Dickinson 740306 T cell Phenotype
CD38 BUV496 HIT2 Becton Dickinson 564658 T cell Phenotype
HLA-DR BUV661 G46-6 Becton Dickinson 565073 T cell Phenotype
CD161 PC7 191B8 Beckman Coulter B30631 T cell Phenotype
CCR6 BV605 G034E3 Biolegend 353420 T cell Phenotype
CCR4 PE-CF594 1G1 Beckton Dickinson 565391 T cell Phenotype
CXCR4 PE 12G5 Biolegend 306506 T cell Phenotype
TBET APC 4B10 Biolegend 644814 T cell Phenotype
GATA3 BV421 16E10A23 Biolegend 653814 T cells Phenotype
CD25 BV785 BC96 BioLegend B302638 T cell Phenotype
CD127 BV650 A019D5 BioLegend B351326 T cell Phenotype
CD95 BUV395 DX2 Becton Dickinson 740306 T cell Phenotype
CD38 BUV496 HIT2 Becton Dickinson 564658 T cell Phenotype
HLA-DR BUV661 G46-6 Becton Dickinson 565073 T cell Phenotype

The dilution for each antibody is specified in Supplementary Table 5.

Validation

All antibodies have been validated by the companies that sell them, in most cases several years ago. All information are available in the website of Becton Dickinson (www.bd.com); Beckman Coulter (www.beckmancoulter.com); Biolegend (www.biolegend.com); ThermoFisher (www.thermofisher.com). We have titrated each of them for the optimal use by flow cytometry, as recommended by the most recent guidelines for the use of cytometry in immunological studies (Cossarizza et al., Eur. J. Immunol. 49:1457-1973, 2019).

Human research participants

Policy information about studies involving human research participants

Population characteristics

A total of 39 COVID-19 patients were included in the study; they had a median age of 64 years (range 35-94), 7 were females, 32 males. Patients were matched for age and gender with a total of 25 healthy donors (CTR), median age 60 years (range 33-66 years). Blood samples (up to 20 mL) were collected in the Infectious Diseases Clinics , brought to the Immunology lab (500 meters away) and immediately processed.

Recruitment

Patients were randomly selected among those who had to start therapy. The group was homogeneous from the clinical point of view, as all of them had confirmed pneumonia.

Ethics oversight

The study was approved by the local Ethical Committee (Comitato Etico dell'Area Vasta Emilia Nord, protocol number 177/2020, March 11th, 2020) and by the University Hospital Committee (Direzione Sanitaria dell'Azienda Ospedaliero-Universitaria di Modena, protocol number 7531, March 11th, 2020). Each participant, including healthy controls, provided informed consent according to Helsinki Declaration, and all uses of human material have been approved by the same Committees.

Note that full information on the approval of the study protocol must also be provided in the manuscript.

Clinical data

Policy information about clinical studies

All manuscripts should comply with the ICMJE guidelines for publication of clinical research and a completed CONSORT checklist must be included with all submissions.

Clinical trial registration

not applicable

Study protocol

This is a case-control, cross sectional, single-centre study

Data collection	All patients entering this study were recruited in the period March 12-20, and April 15-May 10, 2020 in the Infectious Diseases Clinics, University Hospital, via del Pozzo 71, 41124 Modena, Italy.
Outcomes	not applicable

Flow Cytometry

Plots

Confirm that:

- [x] The axis labels state the marker and fluorochrome used (e.g. CD4-FITC).
- [x] The axis scales are clearly visible. Include numbers along axes only for bottom left plot of group (a 'group' is an analysis of identical markers).
- [x] All plots are contour plots with outliers or pseudocolor plots.
- [x] A numerical value for number of cells or percentage (with statistics) is provided.

Methodology

Sample preparation	Analysis of T cell phenotype:<br>PBMC were counted and 500,000 PBMC were stained with the Duraclone IM T cell panel (Beckman Coulter, Miami, FL) added with another five fluorescent mAbs and a marker of cell viability. Beside side and forward scatters, markers were CD45 conjugated with Krome Orange, CD3 APC-A750, CD4 APC, CD8 AF700, CD27 PC7, CD57 Pacific Blue, CD279 (PD-1) PC5.5, CD28 ECD, CCR7 PE, CD45RA FITC, HLA-DR BUV661, CD127 BV650, CD25 BV785, CD95 BUV395, CD38 BUV496, and PromoFluor-840 (Promokine, from PromoCell, Heidelberg, Germany).<br><br>Analysis of intracellular cytokine production:<br>For functional assays on cytokine production by T cells, thawed isolated PBMCs were stimulated for 16 hours at 37°C in a 5% CO2 atmosphere with anti-CD3/CD28 (1 μg/mL) in complete culture medium (RPMI 1640 supplemented with 10% foetal bovine serum and 1% each of l-glutamine, sodium pyruvate, non-essential amino acids, antibiotics, 0.1 M HEPES, 55 μM β-mercaptoethanol). For each sample, at least 2 million cells were left unstimulated as negative control, and 2 million cells were stimulated. All samples were incubated with a protein transport inhibitor containing brefeldin A (Golgi Plug, BD) and previously titrated concentration of CD107a-PE. After stimulation, cells were stained with LIVE-DEAD Aqua (ThermoFisher Scientific, Eugene OR)) and surface mAbs recognizing CD3 PE- Cy5, CD4 AF700, and CD8 APC-Cy7 (Biolegend, San Diego, CA, USA). Cells were washed with stain buffer (BD) and fixed and permeabilized with the cytofix/cytoperm buffer set (BD) for cytokine detection. Then, cells were stained with previously titrated mAbs concentration recognizing IL-17A BV421, TNF BV603, IFN-γ FITC, IL-2 APC, or granzyme-B BV421 (all mAbs from Biolegend).
Instrument	CytoFLEX LX flow cytometer (Beckman Coulter) for T cell phenotype.<br>Attune NxT acoustic cytometer (ThermoFisher) for intracellular staining.
Software	We have used the following softwares:<br>Attune NxT 3.2.1 software (ThermoFisher)<br>Cytexpert 2.4 software (Beckman Coulter)<br>CytoBank (Beckman Coulter)<br>FlowJo software version 9 (Becton Dickinson)<br>R statistical packages (CATALYST 1.10.1). The script is available at: https://github.com/HelenaLC/CATALYST<br>FlowSOM (Bioconductor)<br>Simplified Presentation of Incredibly Complex Evaluation (SPICE) software
Cell population abundance	Aliquots of up to 10 million cells were were thawed immediately before the experiment, and were used only if cell viability was >90%.
Gating strategy	When needed, all Figures report representative examples of gating strategy. There is no supplementary figure since we think that the gating strategy is essential to understand the rest of the figure.

- [x] Tick this box to confirm that a figure exemplifying the gating strategy is provided in the Supplementary Information.
